# (*S*)-1-Ferrocenyl-3-hy­droxy-3-phenyl­propan-1-one

**DOI:** 10.1107/S1600536811000766

**Published:** 2011-01-15

**Authors:** Ping-An Wang

**Affiliations:** a Department of Chemistry, School of Pharmacy, Fourth Military Medical University, Changle West Road 17, 710032 Xi-An, People’s Republic of China

## Abstract

In the title compound, [Fe(C_5_H_5_)(C_14_H_13_O_2_)], the dihedral angle between the phenyl ring and the unsubstituted cyclo­petadienyl ring is 85.0 (2)°while that between the phenyl ring and the substituted cyclo­petadienyl ring is 83.6 (2)°. The dihedral angle between the two cyclo­penta-1,3-diene rings of the ferrocene unit is 2.2 (2)°. The mol­ecules are stabilized by inter­molecular O—H⋯O hydrogen-bonding inter­action within the crystal lattice.

## Related literature

For the preparation, see: Patti & Pedotti (2006*a* ▶); Hashiguchi *et al.* (1995 ▶). For use of the title compound in the preparation of chiral diols, see: Patti & Pedotti (2006*b* ▶) and of phosphine ligands, see: Zhang *et al.* (2007 ▶).
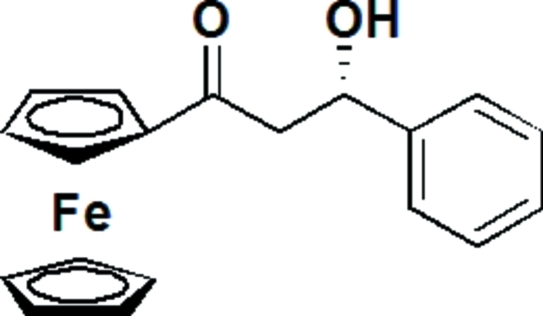


## Experimental

### 

#### Crystal data

[Fe(C_5_H_5_)(C_14_H_13_O_2_)]
*M* *_r_* = 334.18
Orthorhombic, 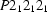

*a* = 10.0609 (14) Å
*b* = 10.6054 (15) Å
*c* = 14.335 (2) Å
*V* = 1529.5 (4) Å^3^
*Z* = 4
Mo *K*α radiation
μ = 0.99 mm^−1^
*T* = 296 K
0.34 × 0.28 × 0.18 mm

#### Data collection

Bruker APEXII CCD diffractometer
Absorption correction: multi-scan (*SADABS*; Bruker, 2005 ▶) *T*_min_ = 0.728, *T*_max_ = 0.840
7586 measured reflections
2721 independent reflections
2424 reflections with *I* > 2σ(*I*)
*R*_int_ = 0.034

#### Refinement

*R*[*F* ^2^ > 2σ(*F* ^2^)] = 0.031
*wR*(*F* ^2^) = 0.066
*S* = 1.06
2721 reflections
200 parameters
H-atom parameters constrained
Δρ_max_ = 0.19 e Å^−3^
Δρ_min_ = −0.22 e Å^−3^
Absolute structure: Flack (1983 ▶), 1146 Friedel pairs
Flack parameter: −0.004 (19)

### 

Data collection: *APEX2* (Bruker, 2008 ▶); cell refinement: *SAINT* (Bruker, 2008 ▶); data reduction: *SAINT*; program(s) used to solve structure: *SHELXS97* (Sheldrick, 2008 ▶); program(s) used to refine structure: *SHELXL97* (Sheldrick, 2008 ▶); molecular graphics: *SHELXTL* (Sheldrick, 2008 ▶); software used to prepare material for publication: *Mercury* (Macrae *et al.*, 2006 ▶) and *ORTEP-3* (Farrugia, 1997 ▶).

## Supplementary Material

Crystal structure: contains datablocks I, global. DOI: 10.1107/S1600536811000766/pb2052sup1.cif

Structure factors: contains datablocks I. DOI: 10.1107/S1600536811000766/pb2052Isup2.hkl

Additional supplementary materials:  crystallographic information; 3D view; checkCIF report

## Figures and Tables

**Table 1:** Hydrogen-bond geometry (Å, °)

*D*—H⋯*A*	*D*—H	H⋯*A*	*D*⋯*A*	*D*—H⋯*A*
O1—H1⋯O2^i^	0.82	2.04	2.841 (3)	166

Symmetry code: (i) 
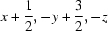
.

**Fig. 1.**
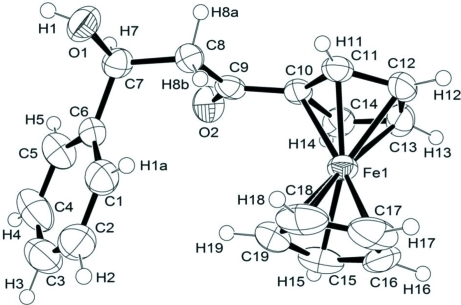
The molecular structure of the title compound, showing the atom-labelling scheme. Displacement ellipsoids are drawn at the 50% probability level. Hydrogen atoms are drawn as spheres of arbitrary radius.

## supplementary crystallographic information

### Comment

The title compound, (*S*)-1-ferrocenyl-3-phenyl-3-hydroxypropane- 1-one, was obtained from the catalytic asymmetric transfer hydrogenation of 1-ferrocenyl-3-phenyl-1,3-dione with the mixed solution of Et_3_N and HCOOH by using Noyori's catalyst (Hashiguchi *et al.* 1995) and it was used for the preparation of chiral diols (Patti *et al.* 2006b) and phosphine ligands (Zhang *et al.* 2007).

In the title compound, C_19_H_18_FeO_2_, the dihedral angle between the phenyl ring and the plane defined by the cyclopetadienyl ring ( C15, C16, C17,C18 and C19)  is 85.0°. However, the dihedral angle between the phenyl ring and the plane defined by the second Cp ring (C10, C11, C12, C13 and C14) is 83.6°. The dihedral angle between the two cyclopenta-1,3-diene rings of the ferrocene moiety is 2.21°. The packing of molecules is stabilized by intermolecular O—H···O hydrogen bonds.

### Experimental

A mixture of [RuCl_2_(</>p-cymene)]_2_ (20.0 mg, 0.030 mmol) and (1</>S,2</>S)-[</>N-(tosyl)- 1,2-diphenylethylendiamine](22.5 mg, 0.061 mmol) in 2-propanol (1.0 cm^3^) was heated at 80°C for 30 min under argon, then the solvent was removed under vacuum. the mixed solution of Et_3_N and HCOOH (1.5 cm^3^) and 1-ferrocenyl-3-phenyl- 1,3-dione (0.34 g, 1.2 mmol) were added to the Ru-complex and the mixture stirred at 50°C while monitoring the reaction progress by thin layer chromatography. After 1.0 h, the solution was diluted with water (3.0 cm^3^) and extracted with ethyl acetate (2×, 20.0 cm^3^). The organic layer was washed with brine (2×, 10.0 cm^3^), dried over Na_2_SO_4_ and taken to dryness under vacuum. The residue was purified on silica gel column to give the title compound (0.31 g, 92% yield), and also produced (1</>S,3</>S)-1-ferrocenyl-3-phenyl- 1,3-dihydroxypropane (11.6 mg, 3.5% yield). The melting point and the spectroscopic data of the title compound were consisted with the reported literature (Patti & Pedotti, 2006a).

### Refinement

All H atoms were placed in calculated positions and refined as riding, with C—H = 0.93–0.98Å and with *U*_iso_(H) = 1.2*U*_eq_(C); with *U*_iso_(H) = 1.5*U*_eq_(O).

### Crystal data

**Table:**

[Fe(C_5_H_5_)(C_14_H_13_O_2_)]	*F*(000) = 696
*M**_r_* = 334.18	*D*_x_ = 1.451 Mg m^−^^3^
Orthorhombic, *P*2_1_2_1_2_1_	Mo *K*α radiation, λ = 0.71073 Å
Hall symbol: P 2ac 2ab	Cell parameters from 2427 reflections
*a* = 10.0609 (14) Å	θ = 2.5–23.9°
*b* = 10.6054 (15) Å	µ = 0.99 mm^−^^1^
*c* = 14.335 (2) Å	*T* = 296 K
*V* = 1529.5 (4) Å^3^	Block, orange-red
*Z* = 4	0.34 × 0.28 × 0.18 mm

### Data collection

**Table:**

Bruker APEXII CCD diffractometer	2721 independent reflections
Radiation source: fine-focus sealed tube	2424 reflections with *I* > 2σ(*I*)
graphite	*R*_int_ = 0.034
φ and ω scans	θ_max_ = 25.1°, θ_min_ = 2.4°
Absorption correction: multi-scan (*SADABS*; Bruker, 2005)	*h* = −8→12
*T*_min_ = 0.728, *T*_max_ = 0.840	*k* = −12→12
7586 measured reflections	*l* = −17→14

### Refinement

**Table:**

Refinement on *F*^2^	Secondary atom site location: difference Fourier map
Least-squares matrix: full	Hydrogen site location: inferred from neighbouring sites
*R*[*F*^2^ > 2σ(*F*^2^)] = 0.031	H-atom parameters constrained
*wR*(*F*^2^) = 0.066	*w* = 1/[σ^2^(*F*_o_^2^) + (0.0253*P*)^2^] where *P* = (*F*_o_^2^ + 2*F*_c_^2^)/3
*S* = 1.06	(Δ/σ)_max_ < 0.001
2721 reflections	Δρ_max_ = 0.19 e Å^−^^3^
200 parameters	Δρ_min_ = −0.22 e Å^−^^3^
0 restraints	Absolute structure: Flack (1983), 1146 Friedel pairs
Primary atom site location: structure-invariant direct methods	Flack parameter: −0.004 (19)

### Special details

**Table:**

Geometry. All e.s.d.'s (except the e.s.d. in the dihedral angle between two l.s. planes) are estimated using the full covariance matrix. The cell e.s.d.'s are taken into account individually in the estimation of e.s.d.'s in distances, angles and torsion angles; correlations between e.s.d.'s in cell parameters are only used when they are defined by crystal symmetry. An approximate (isotropic) treatment of cell e.s.d.'s is used for estimating e.s.d.'s involving l.s. planes.
Refinement. Refinement of *F*^2^ against ALL reflections. The weighted *R*-factor *wR* and goodness of fit *S* are based on *F*^2^, conventional *R*-factors *R* are based on *F*, with *F* set to zero for negative *F*^2^. The threshold expression of *F*^2^ > σ(*F*^2^) is used only for calculating *R*-factors(gt) *etc*. and is not relevant to the choice of reflections for refinement. *R*-factors based on *F*^2^ are statistically about twice as large as those based on *F*, and *R*- factors based on ALL data will be even larger.

### Fractional atomic coordinates and isotropic or equivalent isotropic displacement parameters (Å^2^)

**Table:**

	*x*	*y*	*z*	*U*_iso_*/*U*_eq_	
Fe1	0.11375 (4)	0.33401 (4)	0.18512 (2)	0.03633 (12)	
O1	0.57703 (19)	0.63931 (19)	0.04641 (14)	0.0539 (6)	
H1	0.6135	0.7011	0.0234	0.081*	
O2	0.1578 (2)	0.62907 (17)	0.03540 (14)	0.0533 (6)	
C1	0.4265 (3)	0.6868 (3)	0.2293 (2)	0.0549 (8)	
H1A	0.4579	0.6046	0.2348	0.066*	
C2	0.4006 (4)	0.7564 (4)	0.3096 (2)	0.0698 (10)	
H2	0.4136	0.7208	0.3682	0.084*	
C3	0.3556 (4)	0.8786 (4)	0.3014 (3)	0.0771 (12)	
H3	0.3384	0.9258	0.3548	0.092*	
C4	0.3361 (4)	0.9309 (4)	0.2153 (3)	0.0760 (12)	
H4	0.3062	1.0136	0.2100	0.091*	
C5	0.3611 (3)	0.8600 (3)	0.1359 (2)	0.0572 (9)	
H5	0.3471	0.8956	0.0774	0.069*	
C6	0.4066 (3)	0.7371 (3)	0.14234 (18)	0.0394 (7)	
C7	0.4371 (3)	0.6624 (3)	0.05389 (18)	0.0420 (6)	
H7	0.4081	0.7114	−0.0004	0.050*	
C8	0.3721 (3)	0.5344 (2)	0.05076 (18)	0.0405 (6)	
H8A	0.4048	0.4901	−0.0038	0.049*	
H8B	0.4005	0.4871	0.1052	0.049*	
C9	0.2226 (3)	0.5330 (3)	0.04789 (17)	0.0382 (7)	
C10	0.1570 (3)	0.4105 (3)	0.05858 (18)	0.0364 (7)	
C11	0.2179 (3)	0.2888 (3)	0.06855 (18)	0.0397 (7)	
H11	0.3134	0.2712	0.0657	0.048*	
C12	0.1172 (4)	0.1992 (3)	0.08209 (17)	0.0472 (8)	
H12	0.1309	0.1086	0.0918	0.057*	
C13	−0.0068 (4)	0.2617 (3)	0.08331 (19)	0.0510 (9)	
H13	−0.0936	0.2220	0.0930	0.061*	
C14	0.0165 (3)	0.3913 (3)	0.06844 (19)	0.0460 (8)	
H14	−0.0517	0.4572	0.0656	0.055*	
C15	0.0302 (4)	0.4278 (4)	0.2937 (2)	0.0614 (11)	
H15	−0.0459	0.4851	0.2894	0.074*	
C16	0.0229 (4)	0.2989 (4)	0.3087 (2)	0.0697 (12)	
H16	−0.0583	0.2494	0.3181	0.084*	
C17	0.1545 (5)	0.2528 (4)	0.3103 (2)	0.0751 (13)	
H17	0.1818	0.1652	0.3208	0.090*	
C18	0.2385 (3)	0.3553 (4)	0.2957 (2)	0.0682 (11)	
H18	0.3358	0.3520	0.2932	0.082*	
C19	0.1618 (4)	0.4624 (4)	0.2855 (2)	0.0570 (9)	
H19	0.1950	0.5480	0.2746	0.068*	

### Atomic displacement parameters (Å^2^)

**Table:**

	*U*^11^	*U*^22^	*U*^33^	*U*^12^	*U*^13^	*U*^23^
Fe1	0.0403 (2)	0.0419 (2)	0.02675 (18)	−0.0006 (2)	0.00075 (19)	−0.00136 (19)
O1	0.0498 (13)	0.0496 (14)	0.0624 (13)	−0.0068 (10)	0.0158 (10)	0.0073 (11)
O2	0.0576 (14)	0.0395 (12)	0.0629 (14)	0.0074 (11)	−0.0070 (11)	0.0096 (10)
C1	0.061 (2)	0.056 (2)	0.0474 (17)	−0.0109 (18)	−0.0026 (15)	0.0007 (17)
C2	0.067 (2)	0.098 (3)	0.0443 (19)	−0.024 (2)	−0.005 (2)	−0.002 (2)
C3	0.053 (2)	0.104 (3)	0.074 (3)	−0.011 (2)	0.004 (2)	−0.044 (2)
C4	0.063 (2)	0.065 (2)	0.100 (3)	0.009 (2)	−0.016 (2)	−0.036 (2)
C5	0.058 (2)	0.052 (2)	0.061 (2)	0.0040 (18)	−0.0118 (17)	−0.0081 (16)
C6	0.0406 (18)	0.0392 (15)	0.0385 (15)	−0.0068 (14)	−0.0006 (13)	−0.0025 (12)
C7	0.0494 (17)	0.0373 (15)	0.0392 (14)	0.0004 (15)	0.0046 (13)	0.0062 (15)
C8	0.0461 (17)	0.0339 (14)	0.0414 (15)	−0.0008 (15)	0.0053 (15)	−0.0015 (12)
C9	0.0529 (18)	0.0401 (17)	0.0215 (13)	0.0033 (15)	−0.0007 (13)	−0.0018 (12)
C10	0.0486 (19)	0.0374 (15)	0.0233 (14)	−0.0020 (14)	−0.0010 (13)	0.0013 (12)
C11	0.0526 (19)	0.0377 (17)	0.0289 (15)	0.0038 (15)	0.0048 (14)	−0.0011 (12)
C12	0.074 (2)	0.0366 (15)	0.0312 (14)	−0.0089 (19)	0.0015 (16)	−0.0028 (11)
C13	0.061 (2)	0.057 (2)	0.0347 (18)	−0.0235 (18)	−0.0100 (16)	0.0012 (15)
C14	0.0437 (18)	0.061 (2)	0.0331 (17)	−0.0048 (16)	−0.0093 (15)	0.0033 (15)
C15	0.062 (2)	0.082 (3)	0.040 (2)	0.022 (2)	0.0048 (17)	−0.0185 (18)
C16	0.079 (3)	0.098 (3)	0.0317 (17)	−0.039 (2)	0.018 (2)	−0.007 (2)
C17	0.140 (4)	0.059 (2)	0.0259 (17)	0.028 (3)	−0.006 (2)	0.0069 (18)
C18	0.054 (2)	0.121 (4)	0.0298 (18)	0.008 (2)	−0.0092 (14)	−0.011 (2)
C19	0.072 (2)	0.062 (2)	0.0368 (18)	−0.010 (2)	−0.0026 (15)	−0.0132 (15)

### Geometric parameters (Å, °)

**Table:**

Fe1—C16	2.028 (3)	C7—C8	1.508 (4)
Fe1—C15	2.029 (3)	C7—H7	0.9800
Fe1—C11	2.030 (3)	C8—C9	1.504 (4)
Fe1—C14	2.031 (3)	C8—H8A	0.9700
Fe1—C17	2.032 (3)	C8—H8B	0.9700
Fe1—C10	2.034 (3)	C9—C10	1.466 (4)
Fe1—C18	2.034 (3)	C10—C14	1.435 (4)
Fe1—C19	2.038 (3)	C10—C11	1.436 (4)
Fe1—C13	2.047 (3)	C11—C12	1.402 (4)
Fe1—C12	2.056 (3)	C11—H11	0.9800
O1—C7	1.433 (3)	C12—C13	1.413 (5)
O1—H1	0.8200	C12—H12	0.9800
O2—C9	1.223 (3)	C13—C14	1.410 (4)
C1—C6	1.371 (4)	C13—H13	0.9800
C1—C2	1.392 (4)	C14—H14	0.9800
C1—H1A	0.9300	C15—C19	1.379 (5)
C2—C3	1.377 (5)	C15—C16	1.386 (5)
C2—H2	0.9300	C15—H15	0.9800
C3—C4	1.368 (5)	C16—C17	1.412 (5)
C3—H3	0.9300	C16—H16	0.9800
C4—C5	1.388 (4)	C17—C18	1.393 (5)
C4—H4	0.9300	C17—H17	0.9800
C5—C6	1.385 (4)	C18—C19	1.381 (5)
C5—H5	0.9300	C18—H18	0.9800
C6—C7	1.526 (4)	C19—H19	0.9800
			
C16—Fe1—C15	39.94 (15)	C9—C8—C7	116.3 (2)
C16—Fe1—C11	155.28 (15)	C9—C8—H8A	108.2
C15—Fe1—C11	163.96 (14)	C7—C8—H8A	108.2
C16—Fe1—C14	123.86 (16)	C9—C8—H8B	108.2
C15—Fe1—C14	106.59 (13)	C7—C8—H8B	108.2
C11—Fe1—C14	68.99 (13)	H8A—C8—H8B	107.4
C16—Fe1—C17	40.70 (16)	O2—C9—C10	120.9 (3)
C15—Fe1—C17	67.29 (15)	O2—C9—C8	122.0 (3)
C11—Fe1—C17	121.51 (14)	C10—C9—C8	117.1 (2)
C14—Fe1—C17	162.04 (16)	C14—C10—C11	106.5 (3)
C16—Fe1—C10	161.55 (15)	C14—C10—C9	125.5 (3)
C15—Fe1—C10	125.25 (14)	C11—C10—C9	127.9 (3)
C11—Fe1—C10	41.39 (10)	C14—C10—Fe1	69.21 (17)
C14—Fe1—C10	41.34 (12)	C11—C10—Fe1	69.15 (16)
C17—Fe1—C10	155.97 (17)	C9—C10—Fe1	122.89 (19)
C16—Fe1—C18	67.55 (15)	C12—C11—C10	108.3 (3)
C15—Fe1—C18	66.67 (15)	C12—C11—Fe1	70.94 (16)
C11—Fe1—C18	110.42 (14)	C10—C11—Fe1	69.46 (16)
C14—Fe1—C18	154.93 (16)	C12—C11—H11	125.8
C17—Fe1—C18	40.05 (15)	C10—C11—H11	125.8
C10—Fe1—C18	121.24 (14)	Fe1—C11—H11	125.8
C16—Fe1—C19	67.24 (14)	C11—C12—C13	108.8 (3)
C15—Fe1—C19	39.62 (13)	C11—C12—Fe1	68.93 (15)
C11—Fe1—C19	128.06 (13)	C13—C12—Fe1	69.50 (17)
C14—Fe1—C19	119.70 (14)	C11—C12—H12	125.6
C17—Fe1—C19	67.18 (15)	C13—C12—H12	125.6
C10—Fe1—C19	108.20 (13)	Fe1—C12—H12	125.6
C18—Fe1—C19	39.65 (14)	C14—C13—C12	108.0 (3)
C16—Fe1—C13	106.68 (14)	C14—C13—Fe1	69.16 (19)
C15—Fe1—C13	119.02 (15)	C12—C13—Fe1	70.21 (18)
C11—Fe1—C13	68.29 (12)	C14—C13—H13	126.0
C14—Fe1—C13	40.47 (11)	C12—C13—H13	126.0
C17—Fe1—C13	126.19 (16)	Fe1—C13—H13	126.0
C10—Fe1—C13	68.90 (12)	C13—C14—C10	108.4 (3)
C18—Fe1—C13	164.30 (16)	C13—C14—Fe1	70.37 (19)
C19—Fe1—C13	153.36 (15)	C10—C14—Fe1	69.45 (17)
C16—Fe1—C12	120.49 (13)	C13—C14—H14	125.8
C15—Fe1—C12	154.01 (15)	C10—C14—H14	125.8
C11—Fe1—C12	40.13 (11)	Fe1—C14—H14	125.8
C14—Fe1—C12	67.96 (13)	C19—C15—C16	109.1 (4)
C17—Fe1—C12	109.65 (14)	C19—C15—Fe1	70.6 (2)
C10—Fe1—C12	68.47 (11)	C16—C15—Fe1	70.0 (2)
C18—Fe1—C12	128.76 (15)	C19—C15—H15	125.5
C19—Fe1—C12	165.30 (15)	C16—C15—H15	125.5
C13—Fe1—C12	40.29 (14)	Fe1—C15—H15	125.5
C7—O1—H1	109.5	C15—C16—C17	107.1 (3)
C6—C1—C2	121.2 (3)	C15—C16—Fe1	70.1 (2)
C6—C1—H1A	119.4	C17—C16—Fe1	69.8 (2)
C2—C1—H1A	119.4	C15—C16—H16	126.4
C3—C2—C1	119.4 (3)	C17—C16—H16	126.4
C3—C2—H2	120.3	Fe1—C16—H16	126.4
C1—C2—H2	120.3	C18—C17—C16	107.3 (3)
C4—C3—C2	120.4 (3)	C18—C17—Fe1	70.0 (2)
C4—C3—H3	119.8	C16—C17—Fe1	69.5 (2)
C2—C3—H3	119.8	C18—C17—H17	126.4
C3—C4—C5	119.7 (3)	C16—C17—H17	126.4
C3—C4—H4	120.2	Fe1—C17—H17	126.4
C5—C4—H4	120.2	C19—C18—C17	108.6 (3)
C6—C5—C4	121.0 (3)	C19—C18—Fe1	70.34 (18)
C6—C5—H5	119.5	C17—C18—Fe1	69.9 (2)
C4—C5—H5	119.5	C19—C18—H18	125.7
C1—C6—C5	118.4 (3)	C17—C18—H18	125.7
C1—C6—C7	121.6 (3)	Fe1—C18—H18	125.7
C5—C6—C7	120.0 (2)	C15—C19—C18	108.0 (4)
O1—C7—C8	105.7 (2)	C15—C19—Fe1	69.8 (2)
O1—C7—C6	110.4 (2)	C18—C19—Fe1	70.01 (19)
C8—C7—C6	113.9 (2)	C15—C19—H19	126.0
O1—C7—H7	108.9	C18—C19—H19	126.0
C8—C7—H7	108.9	Fe1—C19—H19	126.0
C6—C7—H7	108.9		
			
C6—C1—C2—C3	−0.8 (5)	C9—C10—C14—Fe1	−116.3 (3)
C1—C2—C3—C4	0.2 (6)	C16—Fe1—C14—C13	−75.3 (3)
C2—C3—C4—C5	0.4 (6)	C15—Fe1—C14—C13	−115.5 (2)
C3—C4—C5—C6	−0.5 (5)	C11—Fe1—C14—C13	80.8 (2)
C2—C1—C6—C5	0.7 (5)	C17—Fe1—C14—C13	−48.3 (6)
C2—C1—C6—C7	178.8 (3)	C10—Fe1—C14—C13	119.5 (3)
C4—C5—C6—C1	−0.1 (5)	C18—Fe1—C14—C13	174.3 (3)
C4—C5—C6—C7	−178.2 (3)	C19—Fe1—C14—C13	−156.5 (2)
C1—C6—C7—O1	−65.9 (4)	C12—Fe1—C14—C13	37.5 (2)
C5—C6—C7—O1	112.2 (3)	C16—Fe1—C14—C10	165.20 (19)
C1—C6—C7—C8	52.7 (4)	C15—Fe1—C14—C10	125.0 (2)
C5—C6—C7—C8	−129.2 (3)	C11—Fe1—C14—C10	−38.73 (18)
O1—C7—C8—C9	−174.2 (2)	C17—Fe1—C14—C10	−167.8 (4)
C6—C7—C8—C9	64.5 (3)	C18—Fe1—C14—C10	54.8 (4)
C7—C8—C9—O2	10.1 (4)	C19—Fe1—C14—C10	84.0 (2)
C7—C8—C9—C10	−171.3 (2)	C13—Fe1—C14—C10	−119.5 (3)
O2—C9—C10—C14	−8.9 (4)	C12—Fe1—C14—C10	−82.0 (2)
C8—C9—C10—C14	172.5 (3)	C16—Fe1—C15—C19	119.8 (4)
O2—C9—C10—C11	176.3 (3)	C11—Fe1—C15—C19	−45.4 (6)
C8—C9—C10—C11	−2.3 (4)	C14—Fe1—C15—C19	−116.8 (2)
O2—C9—C10—Fe1	−95.6 (3)	C17—Fe1—C15—C19	81.1 (3)
C8—C9—C10—Fe1	85.8 (3)	C10—Fe1—C15—C19	−75.3 (3)
C16—Fe1—C10—C14	−42.1 (5)	C18—Fe1—C15—C19	37.5 (2)
C15—Fe1—C10—C14	−74.1 (2)	C13—Fe1—C15—C19	−158.9 (2)
C11—Fe1—C10—C14	118.0 (3)	C12—Fe1—C15—C19	169.6 (3)
C17—Fe1—C10—C14	170.8 (3)	C11—Fe1—C15—C16	−165.2 (4)
C18—Fe1—C10—C14	−156.1 (2)	C14—Fe1—C15—C16	123.4 (3)
C19—Fe1—C10—C14	−114.6 (2)	C17—Fe1—C15—C16	−38.7 (2)
C13—Fe1—C10—C14	37.26 (19)	C10—Fe1—C15—C16	164.9 (2)
C12—Fe1—C10—C14	80.7 (2)	C18—Fe1—C15—C16	−82.4 (3)
C16—Fe1—C10—C11	−160.1 (4)	C19—Fe1—C15—C16	−119.8 (4)
C15—Fe1—C10—C11	168.0 (2)	C13—Fe1—C15—C16	81.3 (3)
C14—Fe1—C10—C11	−118.0 (3)	C12—Fe1—C15—C16	49.8 (4)
C17—Fe1—C10—C11	52.9 (4)	C19—C15—C16—C17	0.4 (5)
C18—Fe1—C10—C11	85.9 (2)	Fe1—C15—C16—C17	60.3 (3)
C19—Fe1—C10—C11	127.47 (19)	C19—C15—C16—Fe1	−59.9 (3)
C13—Fe1—C10—C11	−80.69 (19)	C11—Fe1—C16—C15	170.3 (3)
C12—Fe1—C10—C11	−37.30 (18)	C14—Fe1—C16—C15	−74.6 (3)
C16—Fe1—C10—C9	77.5 (5)	C17—Fe1—C16—C15	117.8 (3)
C15—Fe1—C10—C9	45.5 (3)	C10—Fe1—C16—C15	−42.4 (5)
C11—Fe1—C10—C9	−122.5 (3)	C18—Fe1—C16—C15	80.0 (3)
C14—Fe1—C10—C9	119.6 (3)	C19—Fe1—C16—C15	36.9 (2)
C17—Fe1—C10—C9	−69.6 (4)	C13—Fe1—C16—C15	−115.5 (2)
C18—Fe1—C10—C9	−36.6 (3)	C12—Fe1—C16—C15	−157.1 (2)
C19—Fe1—C10—C9	5.0 (3)	C15—Fe1—C16—C17	−117.8 (3)
C13—Fe1—C10—C9	156.8 (3)	C11—Fe1—C16—C17	52.5 (4)
C12—Fe1—C10—C9	−159.8 (3)	C14—Fe1—C16—C17	167.6 (2)
C14—C10—C11—C12	1.1 (3)	C10—Fe1—C16—C17	−160.2 (4)
C9—C10—C11—C12	176.7 (3)	C18—Fe1—C16—C17	−37.8 (2)
Fe1—C10—C11—C12	60.56 (18)	C19—Fe1—C16—C17	−80.9 (2)
C14—C10—C11—Fe1	−59.4 (2)	C13—Fe1—C16—C17	126.7 (2)
C9—C10—C11—Fe1	116.1 (3)	C12—Fe1—C16—C17	85.0 (3)
C16—Fe1—C11—C12	46.0 (4)	C15—C16—C17—C18	−0.3 (5)
C15—Fe1—C11—C12	−157.1 (5)	Fe1—C16—C17—C18	60.1 (3)
C14—Fe1—C11—C12	−80.3 (2)	C15—C16—C17—Fe1	−60.5 (3)
C17—Fe1—C11—C12	83.4 (3)	C16—Fe1—C17—C18	−118.2 (3)
C10—Fe1—C11—C12	−119.0 (2)	C15—Fe1—C17—C18	−80.2 (2)
C18—Fe1—C11—C12	126.5 (2)	C11—Fe1—C17—C18	84.7 (2)
C19—Fe1—C11—C12	167.8 (2)	C14—Fe1—C17—C18	−153.5 (4)
C13—Fe1—C11—C12	−36.72 (19)	C10—Fe1—C17—C18	46.5 (4)
C16—Fe1—C11—C10	165.0 (3)	C19—Fe1—C17—C18	−37.1 (2)
C15—Fe1—C11—C10	−38.1 (6)	C13—Fe1—C17—C18	169.5 (2)
C14—Fe1—C11—C10	38.68 (18)	C12—Fe1—C17—C18	127.5 (2)
C17—Fe1—C11—C10	−157.6 (2)	C15—Fe1—C17—C16	38.0 (2)
C18—Fe1—C11—C10	−114.5 (2)	C11—Fe1—C17—C16	−157.1 (2)
C19—Fe1—C11—C10	−73.2 (2)	C14—Fe1—C17—C16	−35.3 (6)
C13—Fe1—C11—C10	82.28 (19)	C10—Fe1—C17—C16	164.7 (3)
C12—Fe1—C11—C10	119.0 (2)	C18—Fe1—C17—C16	118.2 (3)
C10—C11—C12—C13	−1.5 (3)	C19—Fe1—C17—C16	81.1 (2)
Fe1—C11—C12—C13	58.18 (19)	C13—Fe1—C17—C16	−72.2 (3)
C10—C11—C12—Fe1	−59.63 (19)	C12—Fe1—C17—C16	−114.3 (2)
C16—Fe1—C12—C11	−159.55 (19)	C16—C17—C18—C19	0.2 (4)
C15—Fe1—C12—C11	165.8 (3)	Fe1—C17—C18—C19	59.9 (2)
C14—Fe1—C12—C11	83.1 (2)	C16—C17—C18—Fe1	−59.8 (3)
C17—Fe1—C12—C11	−115.9 (2)	C16—Fe1—C18—C19	−80.9 (2)
C10—Fe1—C12—C11	38.44 (16)	C15—Fe1—C18—C19	−37.4 (2)
C18—Fe1—C12—C11	−75.0 (2)	C11—Fe1—C18—C19	125.6 (2)
C19—Fe1—C12—C11	−41.2 (5)	C14—Fe1—C18—C19	41.7 (4)
C13—Fe1—C12—C11	120.8 (2)	C17—Fe1—C18—C19	−119.4 (3)
C16—Fe1—C12—C13	79.7 (2)	C10—Fe1—C18—C19	80.8 (2)
C15—Fe1—C12—C13	45.0 (4)	C13—Fe1—C18—C19	−152.1 (5)
C11—Fe1—C12—C13	−120.8 (2)	C12—Fe1—C18—C19	167.19 (19)
C14—Fe1—C12—C13	−37.68 (18)	C16—Fe1—C18—C17	38.4 (2)
C17—Fe1—C12—C13	123.3 (2)	C15—Fe1—C18—C17	81.9 (2)
C10—Fe1—C12—C13	−82.36 (19)	C11—Fe1—C18—C17	−115.1 (2)
C18—Fe1—C12—C13	164.2 (2)	C14—Fe1—C18—C17	161.1 (3)
C19—Fe1—C12—C13	−161.9 (5)	C10—Fe1—C18—C17	−159.8 (2)
C11—C12—C13—C14	1.2 (3)	C19—Fe1—C18—C17	119.4 (3)
Fe1—C12—C13—C14	59.0 (2)	C13—Fe1—C18—C17	−32.8 (6)
C11—C12—C13—Fe1	−57.84 (18)	C12—Fe1—C18—C17	−73.4 (3)
C16—Fe1—C13—C14	123.0 (2)	C16—C15—C19—C18	−0.3 (4)
C15—Fe1—C13—C14	81.5 (2)	Fe1—C15—C19—C18	−59.8 (2)
C11—Fe1—C13—C14	−82.6 (2)	C16—C15—C19—Fe1	59.6 (3)
C17—Fe1—C13—C14	163.4 (2)	C17—C18—C19—C15	0.0 (4)
C10—Fe1—C13—C14	−38.0 (2)	Fe1—C18—C19—C15	59.7 (2)
C18—Fe1—C13—C14	−171.0 (4)	C17—C18—C19—Fe1	−59.7 (2)
C19—Fe1—C13—C14	50.7 (4)	C16—Fe1—C19—C15	−37.2 (2)
C12—Fe1—C13—C14	−119.2 (3)	C11—Fe1—C19—C15	165.5 (2)
C16—Fe1—C13—C12	−117.8 (2)	C14—Fe1—C19—C15	80.0 (2)
C15—Fe1—C13—C12	−159.25 (19)	C17—Fe1—C19—C15	−81.5 (3)
C11—Fe1—C13—C12	36.58 (16)	C10—Fe1—C19—C15	123.7 (2)
C14—Fe1—C13—C12	119.2 (3)	C18—Fe1—C19—C15	−118.9 (3)
C17—Fe1—C13—C12	−77.4 (2)	C13—Fe1—C19—C15	44.7 (4)
C10—Fe1—C13—C12	81.19 (18)	C12—Fe1—C19—C15	−161.9 (4)
C18—Fe1—C13—C12	−51.8 (6)	C16—Fe1—C19—C18	81.8 (2)
C19—Fe1—C13—C12	169.9 (3)	C15—Fe1—C19—C18	118.9 (3)
C12—C13—C14—C10	−0.5 (4)	C11—Fe1—C19—C18	−75.5 (3)
Fe1—C13—C14—C10	59.2 (2)	C14—Fe1—C19—C18	−161.1 (2)
C12—C13—C14—Fe1	−59.7 (2)	C17—Fe1—C19—C18	37.5 (2)
C11—C10—C14—C13	−0.4 (4)	C10—Fe1—C19—C18	−117.3 (2)
C9—C10—C14—C13	−176.1 (2)	C13—Fe1—C19—C18	163.6 (3)
Fe1—C10—C14—C13	−59.8 (2)	C12—Fe1—C19—C18	−42.9 (6)
C11—C10—C14—Fe1	59.4 (2)		

### Hydrogen-bond geometry (Å, °)

**Table:**

*D*—H···*A*	*D*—H	H···*A*	*D*···*A*	*D*—H···*A*
O1—H1···O2^i^	0.82	2.04	2.841 (3)	166

Symmetry codes: (i) *x*+1/2, −*y*+3/2, −*z*.
